# CONGENITAL ANOMALIES OF THE UPPER LIMBS IN A UNIVERSITY CENTER: A CROSS-SECTIONAL STUDY

**DOI:** 10.1590/1413-785220253303e292550

**Published:** 2025-12-01

**Authors:** Danilo José Leite Gomes, Rodrigo Guerra Sabongi, Vinicius Ynoe de Moraes, Luis Renato Nakachima, João Carlos Belloti, Flavio Faloppa

**Affiliations:** 1 Universidade Federal de Sao Paulo, Escola Paulista de Medicina, Departamento de Ortopedia e Traumatologia (EPM-UNIFESP), Sao Paulo, SP, Brazil.

**Keywords:** Clinical Epidemiology, Upper extremity, Congenital abnormalities, Hand Deformities, Epidemiologia Clínica, Extremidade Superior, Anormalidades Congênitas, Deformidades da Mão

## Abstract

**Objective:**

To identify the prevalence and characteristics of CAULs treated at a university center, aiming to improve epidemiological understanding and management of these conditions.

**Methods:**

Study carried out at the Hand Surgery and Microsurgery Service of Hospital São Paulo, UNIFESP, between January 2014 and February 2023. The sample included 296 patients diagnosed with CAULs, whose data were obtained from medical records. Information on sex, type of anomaly, age at first consultation, laterality, time until surgery, number of procedures and consultations were collected. Descriptive statistical analyses and association tests were performed, considering a significance level of 0.05.

**Results:**

It was observed that 61.15% of the patients were from São Paulo, with a higher prevalence of syndactyly (20.3%), followed by preaxial polydactyly (17.9%) and camptodactyly (11.5%). The majority (35.1%) presented bilateral involvement. Conservative treatment was used in 50% of the cases, while surgical intervention, often the only one (34.46%), was necessary in the others.

**Conclusion:**

This study provides an overview of CAULs, with findings that reinforce the predominance of anomalies such as syndactyly and the importance of individualized treatment strategies. *Level of Evidence II; Retrospective* ^f^ *Study.*

## RESUMO

**Objetivo:**

Identificar a prevalência e características das ACMS atendidas em um centro universitário, visando melhorar a compreensão epidemiológica e o manejo destas.

**Métodos:**

Estudo realizado no Serviço de Cirurgia da Mão e Microcirurgia do Hospital São Paulo da UNIFESP, entre janeiro de 2014 e fevereiro de 2023. A amostra incluiu 296 pacientes diagnosticados com ACMS, cujos dados foram obtidos dos prontuários médicos. Foram coletadas informações sobre sexo, tipo de anomalia, idade na primeira consulta, lateralidade, tempo até cirurgia, número de procedimentos e de consultas. Análises estatísticas descritivas e testes de associação foram realizados, considerando um nível de significância de 0,05.

**Resultados:**

Observou-se que 61,15% dos pacientes eram naturais de São Paulo, com maior prevalência de sindactilia (20,3%), seguida de polidactilia pré-axial (17,9%) e camptodactilia (11,5%). A maioria (35,1%) apresentou acometimento bilateral. O tratamento conservador foi utilizado em 50% dos casos, enquanto a intervenção cirúrgica, frequentemente única (34,46%), foi necessária nos demais.

**Conclusão:**

Este estudo fornece um panorama das ACMS, com achados que reforçam a predominância de anomalias como a sindactilia e a importância de estratégias de tratamento individualizadas. *Nível de Evidência II; Estudo Retrospectivo* ^F^.

## INTRODUCTION

Congenital anomalies (CA) are developmental disorders that occur during intrauterine life, affecting the development of the body. They represent a worldwide concern of public health interest, having a significant impact on infant morbidity and mortality.¹ Two clinical causes of congenital anomalies can be observed in the upper limbs: 1) alterations caused during the formation/differentiation process; or 2) alterations induced in the central nervous system.² In Brazil, they are responsible for a considerable percentage of morbidity and mortality in children under one year of age.³ These alterations can be detected during pregnancy or after birth and can be functional (neuromotor), structural (physical deformity) or metabolic (e.g. inborn error of metabolism, phenylketonuria).^4^

The causes of CA can be genetic, environmental and/or multifactorial. Most occur spontaneously or are attributed to genetic factors, while few are associated with teratogens.^4,5^ CAs affect 2 to 3% of live births, and approximately 10% of these children have upper limb abnormalities.^6-8^

Understanding the epidemiology of congenital anomalies of the upper limb (CAUL) is necessary for conducting comparative studies, allowing for the monitoring of changes in the occurrence profile over time, facilitating the development of treatment guidelines.^9-11^ However, knowledge about the presentation and management of these anomalies is still limited in the Brazilian context.^12^

Understanding the profile of patients treated at specialized services for CAUL can have a significant effect on the planning of public policies, monitoring and research in the area. This information contributes to the identification of associated factors, implementation of more effective preventive measures, access to services and, consequently, improvement of health care.

The purpose of this study is to identify the most frequent CAUL and treatment aspects, providing information that can be used to establish strategies that allow for the provision of better care to these patients.

## METHOD

### Study design

This was a single-center, cross-sectional study conducted at the Hand Surgery Department of a university center. This study was approved by the Research Ethics Committee of our institution (number: 5,036,478).

### Sample

We selected, in a convenience sample, all patients treated at the CAUL outpatient clinic between January 2014 and February 2023.

### Inclusion criteria

Individuals of both sexes, regardless of age, diagnosed with CAUL were selected.

### Exclusion criteria

Patients diagnosed with acquired disabilities, deformities caused by tumors, and CA in parts of the body other than the upper limb.

### Data collection

By accessing patient records through the Electronic Medical Record. Information was collected on gender, CAUL diagnoses, age at first consultation, laterality, time since first surgical procedure, number of surgical procedures performed, and number of consultations;

We obtained the data as available in the medical records, and, in their absence, they were considered as lost data.

### Collection instrument

After individual evaluation of the medical records and removal of duplicates, the information was collected and recorded in a spreadsheet in the Microsoft Excel program, version 16.0, developed by Microsoft (Redmond, Washington, United States).

### Statistical analysis

A database was created and, for the analysis of the collected data, descriptive statistics (mean, standard deviation, absolute and relative frequency) were used to characterize the sample studied. Statistical tests were used to assess the association between the variables of interest (sex, most prevalent CA, age at first consultation, location, time until surgical procedure, number of surgical procedures performed and number of consultations).

The results obtained were presented descriptively, in tables and comparative graphs formulated in Excel. Statistical analysis was performed using the Statistical Package for the Social Sciences (SPSS) version 26.0 software developed by International Business Machines Corporation (IBM) (New York, United States) and Minitab Statistical Software version 21.2 developed by Microsoft (Redmond, Washington, United States). A significance level of 0.05 (5%) was set for this study. The mean (μ), median (Md), standard deviation (SD), first quartile (Q1), third quartile (Q3), minimum and maximum values, and confidence interval (CI) were calculated for the number of surgeries, number of consultations, and time until surgery in days.

The Chi-Square or Fisher's Exact Test was used to verify the existence of significant associations between the nominal variables of the study, and the Student's t-test or Mann-Whitney test was used to verify the association between the continuous variables according to the study groups. Associations with a p-value lower than than 0.05 and 95% confidence intervals.

## RESULTS

### Sample characteristics

During the study period, 296 patients with CAUL were treated, 208 surgeries were performed, with an average of 0.7; and 1892 consultations were reached, an average of 6.39 consultations per patient, with a minimum of 1 consultation and a maximum of 39 consultations for the same patient. In addition, the time between the first consultation and the surgical procedure was quite variable, ranging from 6 days to 2024 days, with 75% of the sample having undergone the surgical procedure within 188 days after the first consultation.

Gender: 134 occurred in female individuals (45.3%) and 162 in male individuals (54.7%). When comparing the sexes, it was observed that the number of men was significantly higher than the number of women (p = 0.017)

Place of Birth and Origin: There was a concentration of patients from the city of São Paulo - SP, with 61.15% of the patients being native and 50.68% coming from this location. (Tables 1 and 2).

**Table 1:** Place of birth.

Place of Birth	AF	RF	p
São Paulo - SP	181	61.15%	Reference
Diadema - SP	12	4.05%	0.001*
Taboão da Serra - SP	10	3.38%	0.001*
Others	93	31.3%	0.001*
TOTAL	296	100%	

AF = absolute frequency, RF = relative frequency.

* Statistically significant difference compared to the most frequent place of birth.

**Table 2:** Procedence.

City of Procedence	AF	RF	p
São Paulo - SP	150	50.68%	Reference
Diadema - SP	8	2.7%	0.001*
Embu das Artes - SP	8	2.7%	0.001*
Others	130	43.92%	0.001*
TOTAL	296	100%	

AF = absolute frequency, RF = relative frequency.

* Statistically significant difference compared to the most frequent place of birth.

• Type of Anomaly: Syndactyly emerged as the most prevalent CAUL in the study, accounting for 20.3% of cases. Preaxial polydactyly and camptodactyly followed closely, accounting for 17.9% and 11.5% of cases, respectively (Table 3).
• Location: Most patients (35.1%) presented anomalies in both hands. Unilateral lesions were more frequent in the left hand (29.1%) than in the right (23.3%). In 12.5% of cases, the lesions affected other regions of the upper limb. (Table 4).
• Treatment: Heterogeneity in treatment was observed, with half of the patients being treated conservatively and the other half undergoing surgical procedures. Among the surgical groups, 34.28% underwent a single surgery, while 15.54% required multiple interventions. The comparison between the groups revealed a statistically significant difference in relation to the conservatively treated group (p < 0.001). (Table 5)

**Table 3:** Diagnostic.

Congenital Anomaly	AF	RF	p
Sindactily	60	20.3%	0.006*
Preaxial polydactyly	53	17.9%	< 0.001*
Camptodactyly	34	11.5%	< 0.001*
Congenital trigger finger	30	10.1%	< 0.001*
Postaxial polydactyly	30	10.1%	< 0.001*
Others CA	89	30.1%	Reference
TOTAL	296	100%	

AF = absolute frequency, RF = relative frequency.

* Statistically significant difference compared to the most frequent place of birth.

**Table 4:** Location.

Location of the anomaly	AF	RF	p
Both hands	104	35,1%	Reference
Center hand	86	29.1%	0.113
Right hand	69	23.3%	0.002*
Other locations	37	12.5%	<0.001*
TOTAL	296	100%	

AF = absolute frequency, RF = relative frequency.

* Statistically significant difference compared to the most frequent place of birth.

**Table 5:** Number of surgeries performed.

Number of surgeries	AF	RF	P
None	148	50%	Reference
One	102	34.46%	< 0.001*
Two	36	12.16%	< 0.001*
Three	6	2.03%	< 0.001*
Four	4	1.35%	< 0.001*
TOTAL	296	100%	

AF = absolute frequency, RF = relative frequency.

* Statistically significant difference compared to the most frequent place of birth.

### DISCUSSION

In Brazil, access to specialized outpatient services is provided through regionalized networks and a hierarchy of care, in order to allow users of the Unified Health System (SUS) to access the necessary health services. The fundamental component in the integration of health levels is the referral system (origin, usually Primary Health Care) and counter-referral (specialized service), in which a flow of referrals is carried out between services, considering their different levels of complexity.^13,14^

Congenital and developmental anomalies of the upper limbs are relatively rare. The incidence is 0.2%.^15^ Although CAUL affect relatively few children, the impact on their carriers is usually quite significant. Treatment is complex, with corrective and palliative options that pursue the overall goals of improving the functionality and appearance of affected individuals.^15^

The results of this study corroborate several findings in the literature. The predominance of males, for example, is consistent with observations from previous studies conducted in different geographic contexts.^10-12,16-20^ Despite the higher incidence of CAUL in males, there is little physiological, biological, or endocrinological evidence to explain the phenomenon. One justification raised is that there appears to be a lower frequency of spontaneous abortions of male fetuses with AC, in addition to the action of hormonal factors, which would justify the higher frequency in live-born boys, although this is not true for all types of anomalies.^21^ The high concentration of patients from the capital and metropolitan region of São Paulo highlights the need to expand the supply of centers specialized in CAUL in other regions of Brazil. Centralizing care may hinder access for patients from more remote areas, negatively impacting early diagnosis and appropriate treatment. The higher frequency of syndactyly is in line with findings from studies in the United States,^18^ However, this finding contrasts with the predominance of polydactyly observed in two national studies,^12,20^ in addition to an Australian study,^11^ suggesting a possible influence of regional variations in the distribution of CAUL. Likewise, the high rate of congenital trigger in Swedish^10^ and Japanese^19^ studies contrasts with the lower frequency observed in this study, indicating the need to investigate possible genetic or environmental factors that may influence these differences.

The variability in the time between the first consultation and the performance of the surgical procedure, as well as the high rate of patients who did not undergo surgery, point to the complexity of managing these conditions and the need to individualize the treatment, considering not only the type of anomaly, but also the needs and expectations of each patient^1.0^

Some limitations should be considered: the cross-sectional nature of the study restricts the ability to establish causal relationships between the factors analyzed and the development of CAUL; the retrospective collection of data from medical records may have introduced selection and information biases, limiting the generalization of the results.

Prospective studies with longitudinal patient monitoring are essential to deepen our understanding of the natural history of CAUL, assess long-term treatment outcomes and identify risk factors more accurately.

## CONCLUSION

This study revealed a comprehensive overview of CAUL over a nine-year period. The prevalence and characteristics of CAUL found largely corroborate findings from previous research, reinforcing the importance of some conditions, such as syndactyly and polydactyly.
